# Consumers' Perspectives on National Health Insurance in South Africa: Using a Mobile Health Approach

**DOI:** 10.2196/mhealth.3533

**Published:** 2014-10-28

**Authors:** Edda Weimann, Maria C Stuttaford

**Affiliations:** ^1^School of Public Health and Family MedicineHealth Sciences FacultyUniversity of Cape TownObservatory, Cape TownSouth Africa; ^2^University of ZürichZürichSwitzerland; ^3^University of GöttingenGöttingenGermany; ^4^University of WarwickWarwickUnited Kingdom

**Keywords:** health systems reform, public consultation, South Africa, National Health Insurance (NHI), health systems strengthening (HSS), WHO building blocks, social media, GINI Index

## Abstract

**Background:**

Building an equitable health system is a cornerstone of the World Health Organization (WHO) health system building block framework. Public participation in any such reform process facilitates successful implementation. South Africa has embarked on a major reform in health policy that aims at redressing inequity and enabling all citizens to have equal access to efficient and quality health services.

**Objective:**

This research is based on a survey using Mxit as a mobile phone–based social media network. It was intended to encourage comments on the proposed National Health Insurance (NHI) and to raise awareness among South Africans about their rights to free and quality health care.

**Methods:**

Data were gathered by means of a public e-consultation, and following a qualitative approach, were then examined and grouped in a theme analysis. The WHO building blocks were used as the conceptual framework in analysis and discussion of the identified themes.

**Results:**

Major themes are the improvement of service delivery and patient-centered health care, enhanced accessibility of health care providers, and better health service surveillance. Furthermore, health care users demand stronger outcome-based rather than rule-based indicators of the health system’s governance. Intersectoral solidarity and collaboration between private and public health care providers are suggested. Respondents also propose a code of ethical values for health care professionals to address corruption in the health care system. It is noteworthy that measures for dealing with corruption or implementing ethical values are neither described in the WHO building blocks nor in the NHI.

**Conclusions:**

The policy makers of the new health system for South Africa should address the lack of trust in the health care system that this study has exposed. Furthermore, the study reveals discrepancies between the everyday lived reality of public health care consumers and the intended health policy reform.

## Introduction

In the South African health system, there is a severe divide between the public and private sectors [1]. The proposed National Health Insurance (NHI) scheme aims to bridge existing health inequalities and offer equal access to affordable, quality health care to all citizens, regardless of their socioeconomic status [2]. The escalating gap between the rich and the poor in South Africa is underlined by the Gini index over the last decades (65.0 in 2011 vs 59.3 in 1993) [3,4], which indicates that the disparity is wider than under apartheid [5-7]. The country spends 8.5% of its gross domestic product on its health care system [8], with a poor outcome [9] that is emphasized by a low life expectancy (57 years in males vs 60 years in females) [8] and a high neonatal mortality rate (19 per 1000 live births in 2009) [10-12].

The South African health system is characterized not only by a two-tiered system, but also by escalating costs [13]. The major share of financial and human resources in the health care sector is currently located in the private health sector, which covers only a relatively wealthy minority of the population [2]. While public health care is mainly tax funded and partly by service fees, private health care is financed by privately insured people, medical schemes of public servants, governmental subsidiaries, and public grants. Furthermore, while the costs in the private health sector almost doubled between 1996 and 2003, resulting in increased premiums for private health care users, spending in the public sector decreased [14,15]. Whereas the public annual expenditure is estimated at US $248 per capita, it is US $1002 for the private group [2]. In addition, a major part of public health sector spending is directed towards human immunodeficiency virus/acquired immune deficiency syndrome (HIV/AIDS) and tuberculosis (TB) treatment, to the neglect of other medical areas. The health outcome of people in the private sector differs from the public sector: in 2005 the infant mortality rate (under 1 year) was 4.1 fold higher in the public sector in comparison to the private sector (74 deaths per 1000 live births versus 18 deaths per 1000 live births) [16]. The figure differs across provinces within South Africa and is, for example, 2.6 fold higher in the Eastern than in the Western Cape [17]. Within a decade the maternal mortality rate increased 6.5 fold from 100 deaths in 100,000 pregnancies in 1998 to 650 deaths in 100,000 pregnancies in 2007 [18]. Adding to the decline in the quality of public health services are the poor governance and management of hospitals, public underfunding, mismanagement, shortages of health professionals, and deteriorating infrastructure [1,2,8,19,20]. South Africa also needs to invest in training health professionals, an area that is currently underdeveloped and neglected [19]. The use of measures that optimize efficiency and enable treatment of patients according to their needs [21], such as the triage score [22], are also proposed [23].

The NHI of South Africa seeks to provide universal access to health care, as is promoted by the WHO [24]. Phased in over a period of 14 years, it will lead to major changes in delivery structures, administration, and management systems [2]. These changes are associated with high costs [25], which are estimated at US $33 billion by 2025 [26]. Since different socioeconomic groups have diverse expectations of public health care, South Africans need to be well prepared for major health system changes to ensure their support [20]. Another major concern is the proposed intention to run the health system from the national tier, thus centralizing it [26].

According to Frogner [19], South Africa could reduce the burden of disease by 14.2 million disability adjusted life years (DALYs) and gain up to 184,085 lives by avoiding premature death under a single payer system like the NHI. But this goal can be achieved only if service provision, equity, and efficiency are improved [9]. Currently the NHI is at the Green Paper stage [26], defined as a first-draft document. It is being circulated among interested parties with the intention that they participate in a process of consultation and debate. However, the implementation of the NHI is associated with various logistical and political concerns [1,27]. Hence, it is important to establish public support by reaching and including, as broadly as possible, the opinions of members of the society [28]. Participation can influence service planning, information development, and dissemination as well as the attitudes of service users and providers [29]. It has also been found to have a positive effect on quality and coverage of health care as well as health outcomes [29,30]. As a result, public and patient involvement is increasingly being mainstreamed by various governments [31,32]. It also has the potential to be an important tool for accountability.

A government’s effectiveness is linked to its ability to develop, implement, and enforce measures that ensure the enforcement of policies [32]. This entails the professionalism of the public service, the functioning of governmental departments and agencies, as well as the absence of corruption [33,34]. In addition, it is necessary to establish valid indicators for policy performance [35].

Public consultations not only constitute a civil right in terms of the South African Constitution [36], they may enable enhanced understanding of complex policy changes [37] and can be used to highlight, precisely, the gaps between lived reality and proposed policy [38-40]. The knowledge gained from public consultations can assist [41], as in this case, policy makers to facilitate the overhaul of the health system and to implement the process of a new policy [40,42]. To this end, an electronic consultation process was initiated by a non-governmental organization (NGO) and the People’s Health Movement of South Africa.

The WHO proposes a “building blocks” framework for health systems strengthening (HSS) [43]. The aim of such strengthening is to provide effective, equitable, and high-quality health care as well as to maximize its accessibility [43,44]. Although the WHO building block framework does supply health sector actions for strengthening health systems, the blocks in fact appear static and are not interrelated [45]. Also, the blocks do not specifically address either the role of the population in this process, or the underlying social and economic determinants of health, or the substantial interactions that exist across each component [46]. This is because the building blocks provide an outline for the “hardware” but not for the “software” required to apply ideas and interests, relationships and power, norms [47], values, and human rights to the strengthening process [48]. A practical approach to HSS may, however, be applied through the use of systems thinking [49], which is a means of gaining understanding of the dynamics and the relationships of various stakeholders that would be essential for successful interventions.

The study analyzed the views on a health system policy of consumers—as the system’s major stakeholders. Health systems are meant to be complex adaptive systems that aim to provide improved health as well as social and financial protection as they respond to the expectations and current needs of a population [45]. As a result, in addition to the WHO building blocks, an HSS policy for this country would require the promotion of dynamic networks of diverse stakeholders, the ability to work across subsystems, and the means of inspiring learning and research [49].

Despite consensus on the need of HSS worldwide [50], there is little agreement on which strategies to use in its implementation. The performance of different health systems varies even at the same level of health expenditures or income per capita. These variations are influenced by determinants such as leadership, institutions, system design, and technologies [51].

Public consultation and participation are valuable tools to draw on in support of the successful implementation of new policies and in order to reduce any disparities between intended policies and everyday lived reality. Up to the present, only a few electronic public consultations related to health policies have been published [52]. Governments have not been very active in seeking citizens’ input over the Internet, even though it has become widely accepted by society as a way of conducting everyday life. The advantages of involving the public in decision making are to promote the goals, bind individuals and groups together, support civic and political identity, and create competence and responsibility [53].

Mobile devices that provide social interaction technology applications are ideal for regions such as South Africa with its low Internet and computer penetration, but high coverage on mobile phones [54]. Among social interaction technologies, Mxit is by far the most popular one [55]. More than 6.5 million South Africans run this free instant messaging application on their mobile phones [56-58].Therefore, it was chosen as a tool to reach health care consumers nationwide.

The objective of the study was to understand the experiences and perceptions of public health care users regarding the current health care system and to relate these to the intended reform. The six WHO building blocks were employed as an overarching framework for HSS, and a qualitative research approach was adopted by using a theme analysis to illustrate and interpret the survey data.

This paper outlines health consumers’ views of the current system and whether they are congruent with the proposed improvements of the NHI and the six building blocks for HSS. Since findings may be deployed by policy makers to fine-tune implementation of policy and to fill gaps between public concerns and policy reform, they should facilitate the process of the consumer-orientated overhaul of the health system.

## Methods

Due to the high coverage of mobile phones in South Africa [54], an e-public consultation via Mxit was chosen using mobile devices [55,57]. This public electronic consultation was initiated to raise awareness and facilitate the incorporation of the public’s health needs and concerns into the bill. To ensure the understanding of the participants, short advocacy messages were sent to inform them about the purpose of the consultation and the use of the obtained information to shape the NHI health policy reform. By taking the survey, the participants agreed that their anonymized comments would be used for analysis. Data collection and data analysis were handled separately. All data were rendered anonymously, so that messages could not be traced back to the sender. The study was approved by the Ethics Committee of the University of Cape Town.

The Green Paper was made available on the South Africa National Health Insurance website so as to raise awareness and stimulate public feedback. Mxit donated free advertising for the NHI consultation. An advertisement was sent to 60,000 Mxit users. Nearly 900 participants showed interest in contributing towards the NHI policy. The survey was carried out between November 30 and December 24, 2011. In total, 582 people participated in the survey by submitting answers to the six questions. The questions asked, and their linkage to the six building blocks of the WHO, are illustrated in Table 1. Table 2 displays how many answers were retrieved for each question. In addition, a ranking of the major themes of the survey users was carried out.

**Table 1:** Overview of the questions and statements addressing the South African health care consumers in the survey.

WHO building block	Survey question
Health Service Delivery	The South African Constitution protects the right to health for all people living in SA. Free access to health care services is your right.
Health Workforce	Do you spend hours waiting in line at the clinic every month? South Africa’s public hospitals/clinics need more staff, for example, nurses, doctors, and pharmacists.
Health Information	Prevention is cheaper than treatment! The SA government must provide more health promotion and illness prevention education.
Medicine and Technology	Please give suggestions on how you would like health care services in your community to be improved through the NHI?
Health Care Financing	In SA, over 85% of the population relies on public health care, while only 15% can afford private health care. Yet each sector has almost the same amount of money to spend!
Leadership and Governance	Corruption is a major problem everywhere! How can we prevent corruption from happening in the NHI?

**Table 2:** Semi-quantitative analysis.

Building block and related question	Obtained answers	Ranking of major themes
Service delivery: SA constitution right to free health care	522	1 Good quality of health care provision
		2 Equal health care for all
		2 Treated with respect and dignity
		3 Trust in government
Workforce: waiting times hospitals and shortage of staff	534	1 Waiting time too long
		1 Improve attitude of staff
		2 Improve training of staff
Information: prevention is cheaper than treatment	496	1 Information campaigns (pros and cons)
		2 Affordability of prevention
		3 Better education of people
Medicine and technology: suggestions for improvement	516	1 Improved infrastructure
		1 Improved staff performance
		2 Accessibility of health care providers
Financing: public versus private health care	494	1 Allocation of funds for public health care
		2 Quality of public versus private health care
		2 Affordability of health care
Leadership and governance: Corruption	524	1 Prevention of corruption, law enforcement
		2 Request for reduction of corruption
		3 Introduction of ethical standards for health care professionals
		3 Emphasis on right job qualification

**Figure 1:**
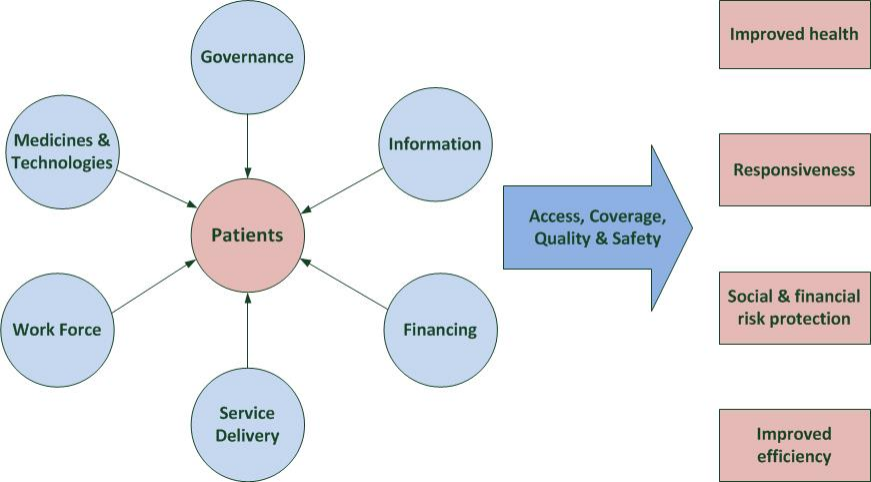
WHO building blocks of a health system [46].

A qualitative analysis [59-61] was performed on the responses. Messages were “cleaned” by deleting space holders and re-allocating the answers to the appropriate questions where necessary. Replies were coded until a saturation of themes was reached, and no new topics emerged. The qualitative data took the form of phrases but were mainly embedded in free-flowing text. Analysis of free-flowing text requires methods that reduce the text to codes [61]. Codes were analyzed by using NVivo as a qualitative data analysis tool, and these were mapped to both NHI themes and the framework of the WHO building blocks (Figure 1). Overall, the dataset should provide a picture of how reform of national health policy is perceived by the public.

Data were analyzed in the light of the research questions: (1) What are the experiences and perceptions of health care users in South Africa?, (2) How would health care users like to see the system improved under the NHI?, and (3) What are the strengths and weaknesses of the WHO building blocks in the light of the answers obtained from the public consultation?

## Results

### Overview

The results are linked to the WHO’s six building blocks of a health system. The consumers’ perspectives are analyzed according to whether the claims of health care users are included in the NHI scheme as planned and if these demands are addressed in the WHO building blocks.

### Health Service Delivery

Health service delivery is a key element in a health care system and a fundamental contributor to the health status of a population [62]. According to the WHO, health service delivery is defined as the delivery and management of safe and quality health services. Resources should be efficiently used and not wasted [63]. The question was phrased as “The South African Constitution protects the right for all people living in South Africa. Free access to health care services is your right”.

The NHI does provide a leadership concept to address the existing inequalities and poor health outcomes in the country as stated by health care consumers in the survey: “Some clinics are dirty and people also suffer from all those germs”, “As South Africans we need to get the right treatment to stay alive”, “They do provide it but the quality is extremely poor”, and “Government must improve health condition in rural areas”. The survey leads the respondents to the matter of human rights, so that some responses read “I have a right for healthy living” or “a healthy country equals to a healthy economy, more jobs are created and less poverty”. Comments such as “the right of health care must be provided by public institutions and not by private ones” or “If it is a free medical services why do we have to pay medical expenses at hospitals?” expressed the view that government is responsible for providing health care for free without charging service fees. Critical voices raise the concern: “What is a right when you are treated with no respect and humanity”, “How reliable is the confidentiality between the patient and the health practitioner?”, and “It is true, but the government still lacks to find people who really care about our health and who do not judge”.

According to the WHO, accountability involves enforcement, such as the imposition of sanctions, the provision of rewards for performance, performance around the actual supply of services, evaluation and monitoring of performance, and financing to ensure that adequate resources are available to deliver essential services [46]. The WHO suggests two types of indicators for measuring governance: rule-based and outcome-based [43]. Rule-based indicators, or so-called formal procedure measurements, are undertaken when a country has appropriate policies, strategies, and approaches for health system governance. The NHI is an approach towards implementing a system based on rule-based indicators. Outcome-based indicators assess whether procedures are being effectively implemented or enforced. The health care users would appreciate the future implementation of outcome-based indicators, although such indicators are not outlined in the NHI. Health care consumers criticize the weak law enforcement and quality control they have experienced, and there is a strong demand from them for better outcome-based rules, monitoring and surveillance; for example, “Quarterly audits might be helpful” and “Have people from department to come and check every day”.

The NHI demonstrates leadership and governance by emphasizing existing inequalities and how to overcome them in a certain timeframe. Yet an existing lack of confidence in the health system is expressed by the respondents: “This statement is our right but I find the government is failing to do this” and “It is a right but everybodies rights are being abused”. This failure of trust will have to be remedied.

### Health Workforce

The workforce is a key element in a health system. It is defined as “people engaged in actions whose primary intent is to enhance health” [43], which includes physicians, nurses, pharmacists, etc. The health workforce is a topic in the NHI and in the WHO building blocks. In the survey, health consumers were asked about waiting times in hospitals and the staffing level of the health workforce.

The analysis of responses revealed that the attitude and training of the health workforce as well as the waiting time are major concerns. The attitude of employees regarding their workplace environment relates to the emotions, level of satisfaction, and their overall outlook. It is often directly related to a high or low level of morale in the workplace. Respondents targeted different disciplines: administration (“The receptionist must always be there”), doctors (“Doctors are more occupied with their own affairs than to treat patients”), and nurses (“The rude treatment [by] the nurses is unethical”). Nurses are more widely discussed, probably because respondents have more intense contact with nurses than with other staff members. The responses express the desire for a more patient-oriented service. From some of the respondents’ points of view, the staff seem to be more focused on their own affairs than on reacting to the patients’ needs.

A good health outcome is largely dependent on the knowledge, motivation, and skills of the health care workforce [45,64], while data support the view that there is a connection between the number of health professionals relative to patients and health outcomes. According to the survey, health care users experience long waiting times in public hospitals but not in private ones. They state that private hospitals have more health professionals available, that they are better trained, better paid, and better motivated to care for people. In addition, they criticize the attitude of the staff in public hospitals: they take breaks that are too long, are absent, are less concerned about their work and the patients, and have been observed “shouting”, “not being empathetic with the patients”, “not been supervised”, and “not well trained”.

A number of responses relate to the training of staff as in the following quote: “Nurses should be trained to take care of patients”. Health care users insist that staff have to receive proper and regular training to fulfill their duties. They also believe that only health workers with a high level of job satisfaction can deliver the best outcome for patients: “The government should keep health workers happy”. Other statements, such as “Health care service is insufficient and [so is] the level of training of health care workers”, suggest that South Africa is not investing enough money to train health professionals and needs to upgrade the quality of service [19]. Since the attitude of the health workforce can be linked to their motivation and work satisfaction, high quality of care cannot be provided unless issues related to demotivated staff are comprehensively addressed [65]. This requires attention if the health system is to be strengthened. Financial incentives, career development, and the quality of management are core factors affecting motivation [66]. Other important elements are adequate resources and appropriate infrastructure [67].

The importance of waiting times for the health care consumers is underscored by the following two quotes: “In public sector patients wait over 12 hours”, and “I never spend less than 4 hours in a clinic”. Different explanations are given for lengthy waiting times: “Long waiting time due to break time for staff”, “The waiting time is too long because the shortage of staff”, and “Long waiting time because everything is free”.

Yet a few respondents report no waiting time. As the data were anonymized, we do not know if this applies to private or public facilities or if the respondents live in a well-serviced area. Waiting times between 4 hours and 12 hours and more are not acceptable to health care users, especially when people are severely sick and urgently need attendance and treatment [68,69]. The government and the individual service providers are asked to reduce these long waiting times and provide quicker help.

Interestingly, health care consumers asked for the implementation of ways to improve treatment efficiency. Separate queues for different diseases are suggested: “for eg influenza in the winter, HIV and TB”. Related to this matter of efficiency, triaging scales are not mentioned as a tool, in the NHI or in the WHO building blocks. Yet they could prove useful in reducing waiting times for severely sick patients and offer more rapid and adequate treatment [23] based on the severity of diseases or illnesses [21]. In countries where they have been introduced, they have been well received by health care consumers and by service providers [22,70]. They would also prove beneficial in South African emergency departments [23].

According to the NHI guidelines, managers should be allocated the necessary authority to achieve planned objectives and should also be held accountable for overall performance and results [2]. Patients report a lack of management and supervision: “Supervise nurses [to stop] long tea breaks”, and “Improve treatment performance by better training”. The respondents complain about insufficient supervision and lack of action taken. In addition, they report waste of resources: “Check how resources are spent and not how much is being spent”.

The attitude of the staff is addressed in the Green Paper of the NHI, where it is portrayed as less service- and patient-oriented than is desirable [2]. Responses in this study also indicate that the level of care is perceived as not centered on the patient’s needs: it is not effective (“received the wrong medication and treatment”) and is not timely (“sometimes I am going home without any treatment and medication”).

Clearly the delivery of health care service through the health workforce needs improvement and scaling up, but it remains traditionally driven, with a paternalistic approach [71]. A shift to an approach more in line with partnership could help to improve service quality [39]. The NHI should foster improvement in levels of trust [72], as well as better relationships between health care consumers and health care providers, and between clinician and patient relationships, in order to improve health outcomes.

The NHI aims to establish a higher quality of service through compliance with the Office of Health Standards: “It will have three units, namely: inspections, norms and standards [...]. It will set norms and standards and undertake the inspection of all health facilities” [2]. However, an area of particular concern is service delivery by the health care workforce [73] and the fact that the NHI plan outlines neither how patient-centered care is to be achieved nor how the regular teaching of health care professionals is going to take place. Based on the responses, neither effective monitoring nor an evaluation of human resources is currently taking place, nor is the assessment of health facilities working adequately. The scaling up of these areas of expertise and service could enhance efficiency as a means of HSS [65].

To estimate if the number of health professionals is adequate, a more thorough analysis must be done by calculating the number of health workers available in a region relative to the total population. The WHO recommends at least 23 health care professionals (physicians, nurses, midwives) per 10,000 people for selected primary health care interventions [46]. Data on the South African health work force in 2011 display 7.7 physicians per 10,000 people and for nursing and midwifery, 40.8 per 10,000 [8]. This exceeds by a total of 48.5 per 10,000 the recommended limit of 23 per 10,000 health care professionals. This fact may be understood to underline the need for better service efficiency, although the uneven distribution of health care professionals between the public and private sector has to be considered.

In the relevant WHO building block, high-quality health services are mentioned, centered on the patient’s need and given in a timely fashion [46]. The way to achieve this is proposed by indicators, mainly related to the amount of available human resources. However, the means of improving interdisciplinary work relations are not elaborated. A more holistic approach to enhanced collaboration between different actors [65] could be helpful in strengthening and improving the health system in South Africa.

### Health Information

The survey question, “Prevention is cheaper than treatment! The South African government must provide more health promotion and illness prevention education”, is linked to the theme of health information. Analysis revealed a number of concerns: affordability (“Prevention should be affordable”), government involvement (“The government is doing enough but the people are ignorant”, “The government is not doing enough”), and the need for a better information policy (“People have less knowledge about prevention”, “The government must try hard to inform the people comprehensively”). Youth are also a matter of concern (“Most of the youth is illiterate”, “Teenagers do not use condoms”).

For people coming from a lower socioeconomic background, resources for obtaining information are scarce. Consequently, they have to rely on whatever information is provided by the municipality or government. Health care users should have access to reliable, usable, understandable, and comparative data and information [43]. They should be informed about health risks so as to avoid contracting diseases. A sound and reliable information policy to support and educate patients is a milestone to establish efficient decision making among the population [46]. The communication and dissemination of information are crucial to an effective prevention campaign. A lack of information or access to relevant information was observable in our study *(*“What is NHI?”), indicating the need for a well-functioning information system [43].

Affordability for the sake of prevention was raised by the respondents. The provision of prevention must be offered based either on the individual’s ability to pay or be free of charge [14]. Further, people need to be informed about preventative measures and strategies. Respondents expressed different views about prevention. Some are convinced that prevention campaigns are successful provided that people are educated (“Education is necessary to run a successful prevention program”, “Teach ignorant people live a healthier life”). Others state that people cannot be taught to adhere to a different lifestyle (“Education does not seem to help. Teens still get pregnant”).

According to the NHI Green Paper [2], prevention campaigns for non-communicable diseases are mainly driven by four risk factors: smoking, alcohol, poor diet, and lack of exercise. Yet multiple other burdens exist [74] that need to be dealt with to improve overall health [73]. Prevention campaigns are associated with various obstacles in South Africa, such as vast rural areas that have poor communication links and a high rate of illiteracy [75].

Participants also conveyed their belief that “the government is doing enough to educate the people”. They point out that a range of prevention programs has been carried out, but people still do not behave accordingly. Hence, they conclude that people cannot be educated and prevention campaigns are a waste of time and resources. Comments like these should sensitize the government to the need to demonstrate the results of prevention campaigns by publishing and advertising data about achieved goals [76].

### Medicine and Technology

The question “Please give suggestions on how you would like health care services in your community to be improved through the NHI” was related to the building block for medicine and technology. Analysis of responses revealed the respondents connecting primary and secondary health care (“24 hours emergency ambulances and emergency rooms”), recognizing intersectoral health care (“Government must provide educated social workers”), and special care (“Employ counsel[l]ors”). They envisage health care through the life course with improved accessibility (“A bus service for pensioners”), besides hospitals’ diverse health care settings (“Provide a nurse at each school”), and improvements in existing services (“ambulances must better work together and be better organized”), and staff performance (“By training the nurses to take good care of patients”, “Would like the nurses to work with their hearts”).

They requested the scaling up of services and medication availability (“Enough medication in hospital is needed”). Even intersectoral cooperation between public and private health care providers was suggested to achieve a better quality of care (“private doctors should work together with normal doctors”).

The maintenance of hospitals and “cleaner hospitals” are frequently mentioned together with the demand for a broad scaling-up initiative of health care facilities. Responses focus on existing clinics (“Better care and longer opening hours”), mobile clinics (“Providing everywhere mobile clinics”), as well as new facilities (“More hospitals are needed because public and private hospitals are full”). Access barriers emerge as another topic: “People in rural areas are not taken care of”, “There is no clinic close to them and there is a lack of water and electricity”, and “A basic health care facility should be in every community”. These demands refer to the need for capacity enhancement [65]. The answers highlight the difficulty the population experiences in gaining access to an adequately equipped health care provider able to answer to their medical needs. According to our survey, the availability and accessibility of health care facilities for public health consumers must be improved.

The respondents expressed wishes similar to those of patients from developed countries, such as the United Kingdom [39]: easier access to primary health care services, more complementary therapies, and longer clinic opening hours. Where there is a contrast between the respondents’ wishes and those expressed in developed countries, it lies in those in the developed countries articulating their desire to have the choice of health service providers, to be actively involved in treatment decision processes, to discuss treatment options, and to have their expectations met [77]. In accordance with UK health care users [39], however, the respondents of our survey expressed their wish to have good and equal services everywhere and available to all.

Most of the public health care consumers’ requests are taken up in the NHI and the WHO building blocks. Under the question “suggestions”, people asked for better access to basic medicines and adequate equipment. Also requested were additional service provisions such as mobile ambulances and better logistics such as the management of ambulance vehicles.

A well-functioning health system ensures equitable access to essential medical products, vaccines, and technologies [46]. The requirements and indicators are outlined in the WHO building blocks. Underpinned by the answers of the respondents, the current SA public health system does not offer equitable access to medical products, vaccines, and technologies. As the data were anonymized, we cannot obtain information about where people report better service quality. But the answers demonstrate that both a lack and uneven distribution of resources appears in rural areas in comparison to urban settlements.

### Health Care Financing

The mismatch of resources between private and public sectors is addressed in the question regarding health care financing and is also elaborated in the Green Paper of the NHI [2]: “In SA, over 85% of the populations rely on public health care, while only 15% can afford private health care. Yet each sector has almost the same amount of money to spend!”.

The consumers surveyed for this study describe the purpose of public health care as follows: “Public health care is important for poor people who cannot afford private health care”. Responses touched on matters of affordability (“A lot of people cannot afford health care”), equality (“If the quality of the health care would be equal, no private health care would be needed”), and funding (“money for the public sector should be reallocated”). The service quality of private health care is regarded as superior to public health care (“People that can afford private health care get better services and are treated better”, "Private health care has a higher standard. So most people do prefer it”, “Public health care has always been a mockery to us because of its poor standards”).

Concerning out-of-pocket payments and service fees*,* some stated that “they must pay in some public clinics”, but others said that “the people in SA get the health service for free”. As suggested by such contradictory answers, the service fees of public health care providers seem not to be handled equally across the country. Apart from the difficulties of accessing health care providers, especially in rural areas, it is essential that lower income groups not be required to make out-of-pocket payments [78]. Financial risk protection is one of the important elements of HSS [65]. The exemption of service fees and the re-engineering of primary health care to improve accessible and affordable health care are proposed in the NHI [2].

The implementation of the NHI should achieve universal coverage, aiming to allow health care users the access to affordable health care services [24,43,79]. A country-wide survey in South Africa that examined household expenditures in relation to out-of-pocket payments showed a regressive profile: the lower the family income, the higher the possibility of experiencing catastrophic household expenditures [15,80]. The NHI Green paper provides an overview of how the new health system will be financed in the near future and funds allocated according to need. The financial system is being conceptually divided into three interrelated functions: revenue collection, fund pooling and purchasing, and provision of services [2]. All three elements were addressed by the responders. They emphasized that they want to experience a more equal and affordable health care system. It is planned to achieve universal coverage through a prepayment health financing mechanism. The payments should be pooled and can come from a combination of sources (fiscus, employers, individuals) [2]. The improvement in resourcing is characterized as an urgent intervention and will be phased in over a period of 7 years. It is estimated that currently 70% of the outpatient and 80% of the inpatient care patients are uninsured [2].

The topic of equality was raised concerning income groups, disease groups (HIV- not HIV-infected), and public and private health care providers having an unequal distribution of financial and human resources. The separation of funds between private and public health care and the contribution to the health care system, depending on personal income are discussed in the survey. Some of the respondents stated that they were not aware of the huge difference in health care expenditures between the private and public sector (“Did not know before how funds are spent”). They requested that people be informed about the existing differences and how to overcome them. Some respondents said that “we all have to get private health care”, as the quality of private health care is considered to be better than in the public sector.

This becomes evident in statements such as “The government should improve the standards for public health care”. Reasons for this, among others, can be seen in the underfunding of public health care: “The public health care does not get enough funds” and the fact that more professionals per patient are working in the private sector than in the public sector [2]. “The amount spent in the private health sector relative to the total number of people covered is not justifiable and defeats the principles of social justice and equity” [2]. Hence a reallocation of funds between the private and public sector is mandatory.

According to the WHO, health financing refers to the “function of a health system concerned with the mobilization, accumulation and allocation of money to cover the health needs of the people [...] to ensure that all individuals have access to effective public health and personal care” [43]. In line with the WHO’s guidelines, co-payments or out-of-pocket payments will be abolished with the NHI. Financial access barriers should be removed. The WHO suggests equity through receiving exemptions or subsidized services and medicines [43]. This should allow people to use needed services without experiencing impoverishment, a method characterized as financial risk protection. Pooled funds are needed where the rich cross-subsidize the poor, and the healthy subsidize the sick population. The respondents target this topic in the following quotes: “Those who can afford more, should pay more” and “Money for the public sector should be reallocated”. It will be one of the major tasks facing the new health system to restructure its financing mechanism and perform a reallocation of funds: the private sector needs to cut back costs, while the funding for the public sector has to be increased to upgrade existing facilities and offer accessible and affordable health care to the majority of the population [2]. It will be a major challenge to achieve the proposed reallocation of funds, and a great deal of lobbying is required to achieve consensus among the different stakeholders [79].

### Leadership and Governance

The WHO, defining the role of the government and the relationship of other actors in order to protect the public interest, describes it as stewardship [43,46]. The existence of strategic political frameworks in combination with effective oversight and accountability are requested [46]. Corruption can impede the delivery of effective and high-quality health care to the people who would benefit most [81]. The World Bank defines corruption as “the abuse of public office for private gain”. Corruption comes in four main types: theft, bribery, misinformation for private gain, and bureaucratic or political corruption [81].

Health care consumers were asked how to prevent corruption in the health system and their responses raised concerns regarding the corruption they are experiencing. They said, for instance, “Reduction of corruption is necessary”*.* Participants proposed solutions to address this problem. Ethical standards should be implemented: “Corruption is unethical. Honesty should be made a value” and “People in high positions must be honest”. In order to achieve such ethical standards, the “right qualifications and experiences” of those working in the health care sector were deemed to be essential. This implies that protocols around hiring staff should be improved. Participants also recommended that “Prevention through work[ing] as a community police force”. And they called for the supervision and punishment of those in charge: “The people in charge should be accountable and the money deducted from their salary”.

According to the study’s results, a new ethical approach for those employed in the health care sector should be introduced, with its stated values demonstrating a high standard of ethical commitment. Some respondents commented that the current state of the health system mirrors the corruption taking place in government: “Prevention by starting at the government”, “Hire qualified people and not politicians”, “Eliminate nepotism and corruption will disappear”, and “Hire people who know the job and are not your relatives...in this corrupt system of government”. A lack of trust in government emerged, thus indicating that it is believed that a change in society would have to take place [33,82] before major improvements would happen in the public health care sector.

The participants asked for the employment and selection of people who were more honest. In order to make people accountable for their actions, they suggested additional audits to expose corruption. The responses display a clear understanding of what actions health care users expect to be taken to address this problem. The majority expressed the feeling that corruption could probably be reduced if the necessary steps were taken but that it cannot be eliminated.

The Green Paper of the NHI does not explicitly address corruption, and consequently, there are no measures described to deal with it. The reform of governance, the autonomy of hospital management as well as overall and individual accountability are mentioned, but outlines on how to provide more efficient supervision of staff and management are missing. Ethical values might be included in the final bill, but it is vital to also incorporate means to reduce corruption, improve surveillance, and to hold individuals to account since corruption is regarded by consumers as a serious problem in the SA health system.

## Discussion

### Principal Findings

The analysis of the answers to the survey answers revealed a public request for improved service efficiency, equity, affordability, and equal allocation of resources between the public and the private sector. These findings substantiate the need for reform and fit with the aims of the NHI. The current state of the health system is described from the patient’s perspective as neither accountable nor efficient. There is a shortage of medicines, uneven distribution of health services, and poor availability of equipment and of intersectoral services. Basic service management appears to be inadequate. The respondents in this study are concerned about the quality of care they are receiving. In general, people judge the quality of care to be better in private hospitals with quicker treatment and less waiting time. The staff in private health care is described as being better organized, more attentive, and more patient oriented in comparison to staff in public health care.

The expectations of health care users are in accordance with those of other countries [39,42,48,77]. Most of the existing and well-documented health inequalities and inefficiencies within the South African population are addressed by the NHI. However, several themes were identified that are not covered, such as the need to fight corruption, the implementation of underlying ethical values for health care professionals, regular surveillance, and indicators for improved health services. These public concerns could be incorporated into the final bill. An enhanced understanding of the goals and timeframe of the NHI should be advertised by the government, as a lack of information to the public becomes evident in the analysis. The NHI, to be implemented gradually over a 14-year period, faces major challenges as various stakeholders are involved in this process. Public health care users perceive the need for changes in the current system, but as is underlined by their comments, they need to understand how these changes are going to happen.

The WHO’s six building blocks identify the key elements of a health system to strengthen health systems. They should lead to improved health, equity, responsiveness, social and financial risk protection, and more efficiency. These building blocks were used as a lens to analyze responses and relate them to the reform of the South African health systems. However, while a health system embraces all organizations, institutions, resources, and people whose primary purpose is to improve health [43], the interactions and interrelations of actors and stakeholders, and measures for improved intersectoral work performance are not elaborated on in the building blocks. Health consumers in South Africa, on the other hand, suggest advancing the intersectoral relationships within the health system. Interestingly, they ask for a more holistic approach and capacity enhancement to establish an efficient working health system [45,65].

The building blocks provide a description of tools that are necessary to strengthen health systems; however, they provide no outline of the soft skills and measures such as values and norms. This is the underlying capital on which a health system runs efficiently and successfully. Each country is asked to implement these soft skills and norms. From the consumers’ perspective, soft skills, interaction techniques, and training of health care professionals need improvement in South Africa. The application of measures for monitoring performance would also prove helpful in enhancing overall performance. Public consultation processes are not often applied in health policy processes, although they constitute a civil right in terms of the South African constitution [36]. The active participation and understanding of the population is needed to restructure the health care system successfully [20]. The health care users in our survey make valuable suggestions as to what can and should be improved in the health system. Up until now, consultation campaigns have not been well-established tools to support health policy changes. The answers obtained could be used by health care decision and policy makers to highlight existing gaps between the lived reality and the set goals, thus aiming to achieve health system strengthening through a patient-centered, outcome-based approach [48].

### Limitations

Some limitations arise regarding the study design: the survey is not representative of the South African population as Mxit is mainly used by a younger population (average 15-35 years) with a different race stratification than in the South African population. For example, there was a higher number of colored people (colored is one of four population groups used by Stats SA, with the others being black African, Indian/Asian, and white): 26% colored people as Mxit users versus 8.9% of the total population. We have no insight into the number of different races that participated in this survey.

### Conclusion

The data obtained in the survey of the People’s Health Movement are useful for further studies and provide insight into the public’s views.These data have been used according to Robson [83] as a real world challenge facing limited time and resources combined with the necessity to address a current problem. These data could be used to highlight existing gaps between the lived reality and the set goals in health care, in order to achieve health system strengthening.

## Abbreviations

DALYs: disability adjusted life years
HSS: health systems strengthening
NGO: non-governmental organization
NHI: National Health Insurance
SA: South Africa
WHO: World Health Organization
